# A Cross‐Tissue Multiomics Analysis Reveals the Protective Role of TGFBR3 in Postmenopausal Osteoporosis

**DOI:** 10.1155/ijog/6364895

**Published:** 2026-04-06

**Authors:** Yimin Liu, Chenxu Xie, Kaiwen Yang, Zixuan Liu, Runtong Liu, Xiaoli Hou, Lei Xing, Jingyuan Gao, Qiangqiang Lian, Yunpeng Hu, Yongheng Wang, Liu Zhang, Faming Tian

**Affiliations:** ^1^ Clinical Medical College, North China University of Science and Technology, Tangshan, 063210, Hebei, China, ncst.edu.cn; ^2^ School of Public Health, North China University of Science and Technology, Tangshan, 063210, Hebei, China, ncst.edu.cn; ^3^ Department of Geriatrics, Affiliated Hospital of North China University of Science and Technology, Tangshan, 063099, Hebei, China; ^4^ Department of Orthopedic Surgery, The Second Hospital of Tangshan, Tangshan, 063000, Hebei, China

**Keywords:** machine learning, Mendelian randomization, postmenopausal osteoporosis, single-cell transcriptomics, TGFBR3

## Abstract

**Background:**

Postmenopausal osteoporosis (PMO) develops as a result of pathological cross‐tissue interactions. However, current experimental paradigms are constrained by their single‐tissue focus, hindering efforts to discover systemwide regulatory genes.

**Objective:**

We aimed to discover conserved genetic regulators of PMO by integrating cross‐tissue transcriptomic profiles in humans and to characterize their biological functions via combined genetic epidemiology and experimental studies using integrated analytical strategies.

**Methods:**

Our analytical framework encompassed transcriptome profiles from human peripheral blood mononuclear cells, bone marrow, and bone tissue. We adopted a tiered strategy involving differential expression analysis, weighted gene coexpression network construction, and machine learning with 108 algorithm combinations for candidate gene selection. A two‐sample Mendelian randomization was used to inform causal gene–disease relationships, while the key results were validated in an ovariectomized mouse model of osteoporosis. Mechanistic studies included single‐cell transcriptomics, functional enrichment, and immune microenvironment profiling.

**Results:**

Cross‐tissue analysis identified 97 consistently dysregulated genes between tissues, which were further refined to 64 high‐confidence candidates. TGFBR3 was significantly protective against PMO (IVW OR = 0.675, 95% CI: 0.466–0.977, *p* = 0.037). Osteoporotic mice exhibited considerable downregulation of TGFBR3 expression, which was positively correlated with bone mineral density and mechanical properties as well as bone formation markers and negatively correlated with resorption markers. Cellular localization showed enrichment of TGFBR3 in bone marrow mesenchymal stem cells and T cells from human and mouse bone marrow. Functional analyses suggested that its protective effects involve the modulation of osteogenic differentiation pathways and regulation of the immune microenvironment.

**Conclusion:**

This is the first study to identify TGFBR3 as a novel cross‐tissue protective regulator of PMO. Our integrated approach covering genomic discovery, causal inference, and experimental validation offers strong support to the hypothesis that TGFBR3 deficiency constitutes a fundamental feature of PMO pathogenesis, while shedding light on its multilevel protective mechanisms.

## 1. Introduction

Postmenopausal osteoporosis (PMO) is a highly prevalent metabolic skeletal disorder, commonly affecting women after menopause, with a significant impact on public health [1, 2]. Elucidating the molecular basis of PMO and identifying novel therapeutic targets hold significant scientific and clinical relevance.

Skeletal homeostasis is maintained through integrated signaling between the local bone tissue and the bone marrow microenvironment on the one hand and systemic immune pathways on the other hand, thus constituting a sophisticated osteoimmune network [3–6]. Although these physiological units are highly functionally dependent on each other, current experimental protocols typically study them in a segregated manner. This segregation has precluded the identification of conserved regulatory genes acting across tissue boundaries and thus fundamentally limits our understanding of PMO pathogenesis [7–9].

New methodological tools provide novel opportunities for comprehensive disease studies [10, 11]. Machine learning approaches enable efficient extraction of biologically relevant features from complex genomic datasets [12–14]; Mendelian randomization (MR) utilizes genetic instrumental variables (IVs) to infer causal relationships between genes and diseases, mitigating confounding biases inherent in traditional observational studies to some extent [15–17]. Finally, single‐cell transcriptomics further allows for a novel level of resolution in mapping gene expression patterns across a variety of cellular populations [18, 19].

Accordingly, this study proposes an integrated analytical strategy: candidate genes are screened through cross‐tissue transcriptome integration, followed by employing machine learning and MR to identify the key causal gene, TGFBR3. Subsequently, animal experiments are conducted to validate the association between its expression and bone phenotypes. Finally, single‐cell and functional analyses are leveraged to preliminarily explore its potential mechanisms of action, aiming to provide new evidence for target discovery in PMO.

## 2. Materials and Methods

### 2.1. Cross‐Tissue Transcriptomic Data Sources

Microarray and bulk RNA‐seq datasets GSE230665, GSE35956, GSE56814, GSE56815, and GSE56116 were included; specifically, GSE230665 provides RNA‐seq data from femoral head tissue of 12 PMO patients and 3 healthy postmenopausal controls, GSE35956 originally contained expression profiles from 10 human bone marrow mesenchymal stem cell samples, and after excluding one male sample, four healthy postmenopausal controls and five osteoporotic women were retained [20]; both GSE56814 and GSE56815, based on the Affymetrix platform, profiled female peripheral blood mononuclear cells and, after postmenopausal stratification, provided data from 26 high hip bone mineral density and 16 low hip bone mineral density individuals, and 20 high hip bone mineral density and 20 low hip bone mineral density individuals, respectively [21, 22]; GSE56116 includes RNA‐seq data from peripheral blood mononuclear cells of 10 PMO patients and three healthy postmenopausal controls, used for assessing systemic inflammatory signals [23].

Specifically, GSE230665 was utilized as the single representative dataset for femoral tissue and GSE35956 for bone marrow tissue, while the mononuclear cell group consisted of a combined dataset from GSE56814, GSE56815, and GSE56116.

### 2.2. Processing of Cross‐Tissue Transcriptomic Data

Transcriptomic datasets underwent normalization using the “limma” package. To address technical variability, datasets were integrated after batch effect correction with the “sva” package. The efficacy of batch removal was confirmed via principal component analysis (PCA) on the standardized combined dataset. Samples were classified into osteoporosis (OP) and control groups according to their original metadata. Finally, differential expression analysis was carried out with “limma,” applying thresholds of |logFC| > 0 and an adjusted *p* value of < 0.05 to define significantly dysregulated genes.

### 2.3. Machine Learning for Model Screening and Evaluation

For machine learning analysis, the integrated dataset from GSE230665, GSE35956, GSE56814, GSE56815, and GSE56116 functioned as the training set, with each constituent dataset serving independently for validation. We implemented a diverse set of ten algorithms—LASSO [24], Ridge [25], elastic net (Enet) [26], random forest (RF) [27], Stepglm [28], generalized boosted regression modeling [29], SVM [30], XGBoost [31], glmBoost [28], and naive Bayes [28]—applying them to the MetaGSE dataset. Feature selection and model construction were performed using tenfold cross‐validation. Model performance was assessed by calculating the area under the curve (AUC) and 95% confidence intervals (CIs) for both training and validation sets. Following AUC‐based ranking of all models, the most suitable model was identified through ROC analysis executed with the pROC package.

### 2.4. SHapley Additive exPlanations (SHAP) Analysis for Interpreting Feature Contributions of the Selected Model

SHAP values were utilized to quantify the predictive contribution of key features associated with OP development. This approach allowed for analyzing the relative importance of individual features in outcome prediction and visualizing the contribution of each significant feature to the final machine learning model’s output.

### 2.5. MR Data

The expression quantitative trait locus (eQTL) data used in the MR analysis were obtained from the eQTLGen consortium (https://eqtlgen.org/), comprising cis‐eQTLs for 16,989 genes derived from 31,684 blood samples of healthy individuals of European ancestry [32]. A comprehensive overview of this dataset is provided in the original publication [32]. The outcome data were sourced from the finngen_R12_M13_OSTEOPOROSIS phenotype in the FinnGen Release 12, published in September 2023 (https://www.finngen.fi/). Detailed descriptions of the FinnGen data are available on the official website (https://r12.finngen.fi). All contributing genomewide association studies included in these resources were conducted in accordance with ethical standards.

### 2.6. Two‐Sample MR

A two‐sample MR analysis was performed using eQTLs as the exposure and finngen_R12_M13_OSTEOPOROSIS as the outcome. Because both the eQTLGen consortium and FinnGen database include individuals of European ancestry, there may be a sample overlap between the exposure and outcome datasets. However, the inverse variance weighted (IVW) method used in our main analysis has been shown to be reasonably robust to bias from the sample overlap, unless the overlap is extreme. We also used supplementary methods (MR‐Egger and weighted median), which offer alternative causal estimation approaches. Single‐nucleotide polymorphisms (SNPs) significantly associated with the exposure at the genomewide significance level (*p* < 5 × 10^−8^) were selected as potential IVs. PLINK 1.9 was used to prune SNPs in linkage disequilibrium (LD) using a clumping window of 10,000 kb and an *R* ^2^ threshold of < 0.001, ensuring the independence of the IVs [16]. SNPs with a minor allele frequency (MAF) ≥ 0.01 were retained, and weak instruments (F‐statistic < 10) were excluded [33]. The IVW method was used as the primary analysis for estimating the causal effect. Heterogeneity was assessed using Cochran’s *Q* test; a random‐effects IVW model was applied if *p* < 0.05; otherwise, a fixed‐effects model was used. To ensure the robustness of the results, three key assumptions were simultaneously evaluated: (1) the MR‐Egger regression intercept test *p* > 0.05, indicating no significant directional pleiotropy [34]; (2) the direction of effect estimates from the IVW, weighted median, and MR‐Egger methods were consistent (i.e., the same sign for the β coefficient); and (3) the MR‐PRESSO global test *p* > 0.05, and the IVW result remained statistically significant after neither detecting nor removing outlier SNPs (outlier threshold *p* < 0.05, NbDistribution = 3000, SignifThreshold = 0.05) [16]. Additionally, a leave‐one‐out sensitivity analysis was conducted to identify any potential outlier effects driven by a single SNP. All analyses were performed in *R* Version 4.3.2 using the TwoSampleMR (Version 0.5.6) and MR‐PRESSO (Version 1.0) packages [16].

It is important to note that our MR analysis is based on the following three IV assumptions:

1. Relevant assumption: The selected genetic variants are highly correlated with TGFBR3 expression.
2. Independence assumption: The selected genetic variants are independent of any known or unknown confounders of the TGFBR3–OP association.
3. Exclusion restriction assumption: The selected genetic variants affect OP risk only through their effects on TGFBR3 expression and not through other biological processes.

Additionally, as we conducted an exploratory study on the top 10 feature genes identified by machine learning, we did not perform multiple testing correction on the MR analysis. The primary goal of this study was to find potential causal relationships that could be further tested by experiments, rather than obtaining statistical significance.

All summary‐level data and the *R* code generated during the MR analysis are available from the corresponding author upon reasonable request. This MR study was conducted strictly in accordance with the necessary steps of the STROBE‐MR guidelines, and the completed STROBE‐MR checklist is provided in Supporting File 1.

### 2.7. Animal Experimental Grouping

All animal procedures were approved by the Animal Ethics Committee of North China University of Science and Technology (Approval No. SQ2024142). Twelve 14‐week‐old specific pathogen‐free (SPF) female C57BL/6J mice were randomly assigned to either a sham‐operated group or a bilateral ovariectomy (OVX) group (*n* = 6 per group). The animals were housed in the SPF facility of North China University of Science and Technology under a controlled environment (temperature: 22 ± 2°C, humidity: 50%–60%) with a 12‐h light/dark cycle. All mice were given free access to sterile water and standard pellet diet. Nesting materials were provided in each cage as environmental enrichment to enhance animal welfare. The health status of the animals was monitored daily, and humane endpoints (e.g., > 20% weight loss, prolonged lethargy, or severe infection) were established, although no animals reached these endpoints during the study. Mice in the OVX group underwent bilateral ovariectomy under anesthesia to induce an OP model, while sham mice underwent the same surgical procedure without ovary removal. Following surgery, mice were maintained until 22 weeks of age. At the end of the experimental period, mice were euthanized by an intraperitoneal overdose of pentobarbital sodium (200 mg/kg) followed by cervical dislocation to ensure death, and bilateral femurs and tibiae were harvested for subsequent analyses.

### 2.8. Assessment of Femoral Bone Microarchitecture by Microcomputed Tomography (Micro‐CT)

Right femurs and tibiae (*n* = 6 per group) from 22‐week‐old mice were scanned using high‐resolution micro‐CT. To quantify the bone microarchitecture and ensure anatomical consistency across samples, the bone region of interest (ROI) was precisely defined using standardized anatomical landmarks. To exclude the primary spongiosa and focus on the mature trabecular compartment, the starting point (offset) for the proximal tibial cancellous ROI was set at the distal boundary of the growth plate. From this starting point, the ROI extended toward the diaphysis for a distance equivalent to 5% of the total tibial length (approximately 1.0 mm), which was designated as the volume of interest (VOI). For cortical bone analysis, the ROI was defined as a 1.0‐mm segment centered at the tibial midpoint (calculated as 50% of the total bone length, extending 0.5 mm both proximally and distally). The following parameters were analyzed and reported with their respective units: bone mineral density (BMD, g/cm^3^), bone volume fraction (BV/TV, %), structure model index (SMI, dimensionless), cortical bone thickness (Ct.Th, mm), cortical bone area fraction (Ct.Ar/Tt.Ar, %), trabecular number (Tb.N 1/mm), trabecular separation (Tb.Sp, mm), and trabecular thickness (Tb.Th, mm) [35].

### 2.9. Three‐Point Bending Test of the Femur

To evaluate the biomechanical properties of the bone (*n* = 6 per group), the left femurs were subjected to a three‐point bending test using a material testing machine. Each femur was positioned on two metal support bars with a fixed span length of 6 mm, ensuring the patellar surface faced upward. A constant vertical compressive load was applied to the midpoint of the femur at 2 mm/min until fracture occurred. The following biomechanical parameters were calculated and recorded using the system’s integrated software: maximum load (N), maximum deflection (mm), maximum stress (N/mm^2^), stiffness (N/mm), elastic load (N), elastic deflection (mm), elastic stress (N/mm^2^), elastic modulus (E, N/mm^2^), and elastic energy absorption (N·mm) [36].

### 2.10. Western Blotting

Western blotting was performed following standard procedures. Briefly, frozen bone tissues were pulverized into a fine powder in liquid nitrogen. Total proteins were then extracted by lysing the tissue powder in RIPA buffer supplemented with protease and phosphatase inhibitors [37, 38]. To ensure complete denaturation and solubilization of proteins, particularly for the effective recovery of membrane‐bound (TGFBR3 and receptor activator of nuclear factor kappa‐B ligand [RANKL]) and secreted proteins (osteoprotegerin [OPG] and osteocalcin [OCN]), the lysate was briefly pipetted and incubated at 100°C for 5 min. After centrifugation, the protein concentration in the supernatant was determined using the BCA assay. Proteins were separated by SDS–PAGE with a 10% separating gel and transferred onto a PVDF membrane. After blocking with 5% nonfat milk, the membranes were incubated overnight at 4°C with the following primary antibodies: anti‐OPG (1:1000, Proteintech, China, A00863), anti‐OCN (1:1000, Abbkine, China, A20800), anti‐RANKL (1:1000, Proteintech, China, 66610‐1‐Ig), anti‐GAPDH (1:1000, Abcam, UK), and anti‐TGFBR3 (1:1000, ABclonal, China, A0627). Subsequently, the membranes were incubated with horseradish peroxidase (HRP)‐conjugated secondary antibodies for 2 h at room temperature. Protein bands were visualized using an ECL detection reagent and quantified by grayscale analysis using ImageJ software.

To ensure the accuracy of fold‐change comparisons, a dual‐normalization strategy was applied: first, the grayscale intensity of each target protein was normalized to its respective GAPDH internal control; second, the resulting ratios were scaled to the mean value of the control (Ctrl) group. This procedure ensured that the mean relative protein expression of the Ctrl group was centered at 1.0, enabling a clear visualization of relative fold changes.

### 2.11. Quantitative Real‐Time Polymerase Chain Reaction (qRT‐PCR) Analysis

Total RNA was extracted from bone tissues using the TRIzol method with liquid nitrogen grinding. RNA concentration and purity were measured, and samples with an A260/A280 ratio between 1.8 and 2.0 were used for subsequent analysis. cDNA was synthesized by reverse transcription and stored at −20°C. qRT‐PCR was performed according to the manufacturer’s instructions. The relative expression levels of target genes were normalized to GAPDH and calculated using the 2−ΔΔCt method. The primer sequences used were as follows: OPG, forward: CAC​AGT​GAG​GAG​GAA​GAC​ATT, reverse: GAG​AAG​AAC​CCA​TCT​GGA​CAT; RANKL, forward: GAC​GTT​AAG​CAA​CGG​AAA​ACT​AA, reverse: GCT​AAT​GTT​CCA​CGA​AAT​GAG​TC; OCN, forward: GGA​CCA​TCT​TTC​TGC​TCA​CTC​TGC, reverse: TCC​TGC​TTG​GAC​ATG​AAG​GCT​TG; TGFBR3, forward: TGG​ACC​TGG​TCA​ACA​ACT​ACG, reverse: AGG​TAG​GCG​TTG​TCC​TTC​AG and GAPDH, forward: GAC​AAC​TTT​GGC​ATT​GTG​GA, reverse: ATG​CAG​GGA​TGA​TGT​TCT​GG.

### 2.12. Single‐Cell Analysis

Single‐cell data included GSE147287 and GSE253355. The former dataset was derived from femoral heads of two patients undergoing total hip arthroplasty for the end‐stage hip disease, where fresh isolated CD271^+^ bone marrow mononuclear cells underwent single‐cell transcriptome sequencing; one patient was diagnosed with PMO, while the other served as an osteoarthritis control [39]. The latter dataset was collected from bone marrow samples of 12 patients with normal hematological parameters undergoing total hip arthroplasty, used to construct a reference atlas of the bone marrow microenvironment [40].

The complete analytical pipeline for single‐cell transcriptomic data was implemented in *R* Version 4.3.2 using Seurat v5. Following the official 10 × Genomics Cell Ranger v7.1.0 workflow, the GRCh38‐2020‐A reference genome was used for alignment and quantification, generating a filtered feature‐barcode matrix for each sample. Uniform quality control criteria were applied: cells with gene counts between 200 and 6,000, UMI counts between 500 and 40,000, and mitochondrial gene percentage < 10% were retained to remove low‐quality cells and potential doublets. Following standard quality control procedures, the single‐cell data were processed using the SCTransform normalization method, which accounted for variations in total UMI counts and mitochondrial gene content. We then identified the 3000 most variable genes for subsequent analytical steps, including dimensionality reduction and cell clustering.

### 2.13. Functional Enrichment Analysis

We first stratified samples into TGFBR3 high‐ and low‐expression cohorts based on median expression levels. Gene set variation analysis (GSVA) was subsequently performed to transform transcriptomic profiles into pathway activity matrices. Additionally, gene set enrichment analysis (GSEA) was conducted as a complementary approach using the same expression‐based grouping strategy. Pathway enrichment scores from GSVA were calculated separately for each expression group, while GSEA evaluated the enrichment of predefined gene sets. Differential pathway activity was defined as |log_2_FC| > 0 with FDR < 0.05 for GSVA results, whereas GSEA significance required FDR < 0.05 and |NES| > 1. Statistical evaluation of differential activities between cohorts was carried out employing the limma package [41–43].

### 2.14. Statistical Analysis

Statistical analysis was performed using IBM SPSS Statistics software (Version 23.0; IBM Corp., Armonk, NY, USA). We give continuous variables as mean ± standard deviation. When making group‐to‐group comparisons, appropriate statistical tests were chosen based on data distribution aspects. The independent Student’s *t* test was used for comparing two groups when the assumptions of normal data and equal variance were met; otherwise, the Mann–Whitney *U* test was adopted. One‐way ANOVA was used for multigroup comparisons, and the LSD post hoc test was applied for pairwise comparisons if the ANOVA result was significant (*p* < 0.05). The relationship between variables was measured via Pearson correlation analysis. All reported *p* values, being two‐sided, have *p* < 0. One was regarded as statistically significant. Figures’ error bars portray mean ± SD. Specific significance levels in the figures are denoted as ^∗^ *p* < 0.05, ^∗∗^ *p* < 0.01, and ^∗∗∗^ *p* < 0.001, with exact *p* values reported in the figure legends. Results meeting the statistical threshold of *p* < 0.05 are the only ones for which the term “significant” is strictly reserved.

During the data analysis, the investigators were blinded to the group allocation to ensure the objectivity of the results. Furthermore, this manuscript was prepared strictly in accordance with the journal’s submission requirements, and the author checklist of the required items to address in compliance with the submission and formatting guidelines is provided in Supporting File 2.

## 3. Results

### 3.1. Identification of Shared Differentially Expressed Genes (DEGs) in Cross‐Tissue Transcriptomic Datasets

To identify genes commonly dysregulated in bone marrow (GSE35956), femoral tissue (GSE230665), and circulating mononuclear cells, transcriptomic datasets GSE56814, GSE56815, and GSE56116 derived from human peripheral blood mononuclear cells were merged. After batch correction and renormalization, a “combined mononuclear cell dataset” was established. Standardized boxplots (Figure 1(a)) and PCA plots (Figure 1(b)) both demonstrated effective control of batch effects. Differential expression analysis was performed separately on the combined mononuclear cell dataset, bone marrow tissue, and femoral tissue, with results displayed in volcano plots (Figure 1(c)). Subsequently, upregulated and downregulated genes from each of the three tissue groups were extracted and intersected. Venn diagram analysis identified a total of 97 shared DEGs (Figure 1(d)).

FIGURE 1Workflow and results of cross‐tissue transcriptomic differential analysis. (a) Standardized boxplots of each dataset after batch correction and renormalization. (b) Principal component analysis plot. (c) Volcano plots displaying differentially expressed genes in the combined mononuclear cell dataset, bone marrow, and femoral tissue (screening criteria: |log_2_FC| ≥ 0 and *p* < 0.05). (d) Venn diagram showing the intersection of upregulated and downregulated genes from the three tissue sources.

**Figure:**
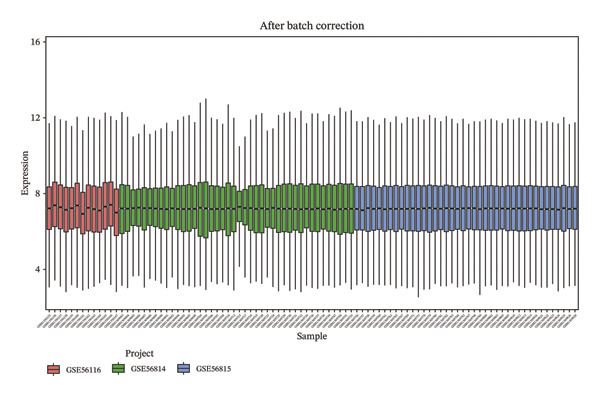
(a)

**Figure:**
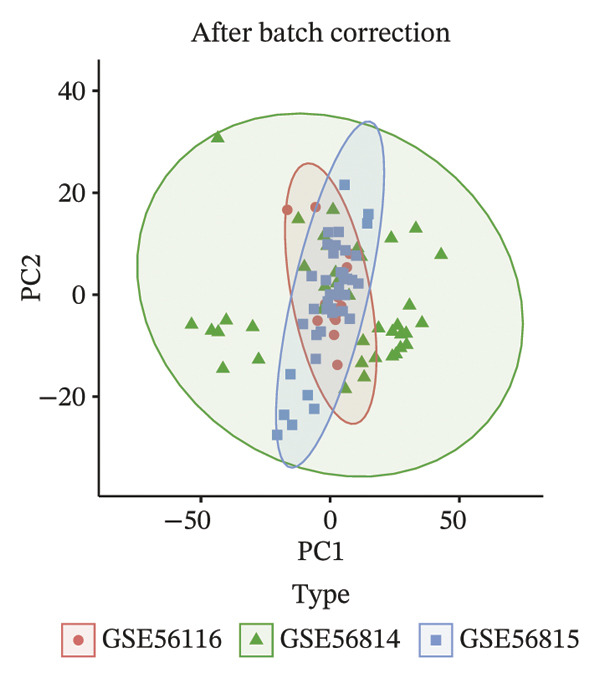
(b)

**Figure:**
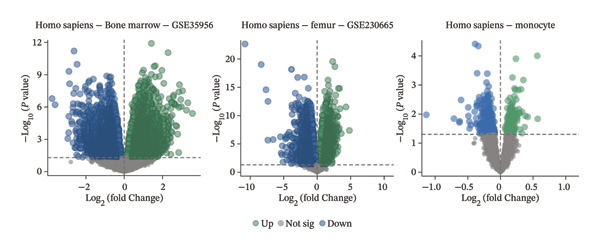
(c)

**Figure:**
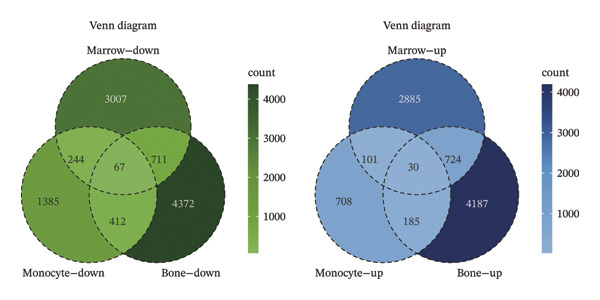
(d)

### 3.2. Construction of Cross‐Tissue Coexpression Networks and Identification of PMO‐Related Modules

Transcriptomic datasets from human peripheral blood mononuclear cells (GSE56814, GSE56815, and GSE56116), bone marrow tissue (GSE35956), and femoral tissue (GSE230665) were batch‐corrected and renormalized and then merged to construct a “cross‐tissue combined dataset.” A comparison of PCA plots before (Figure 2(a)) and after integration (Figure 2(b)) showed that batch effects were effectively eliminated. WGCNA was performed on this combined dataset. Through scale‐free topology fitting analysis, a soft threshold of 10 (*R* ^2^ > 0.9) was determined to construct a scale‐free coexpression network (Figure 2(c)), which also met the requirement for stabilized mean connectivity (Figure 2(d)). Based on this, all genes were divided into 10 coexpression modules, and a module–trait relationship heatmap was generated. The results showed that the MEgrey, MEpink, MEmagenta, MEbrown, MEred, and MEblack modules were significantly associated with the PMO phenotype, all with statistical significance (Figure 2(e)).

FIGURE 2Workflow and results of cross‐tissue coexpression network analysis. (a) Principal component analysis plot of human PBMC, bone marrow, and femoral tissue transcriptomic data before batch correction and renormalization. (b) Principal component analysis plot after batch correction and renormalization. (c) Scale‐free topology fitting analysis for determining the soft threshold. (d) Analysis of mean connectivity under different soft thresholds. (e) Module–trait relationship heatmap displaying correlations between identified coexpression modules and PMO phenotype.

**Figure:**
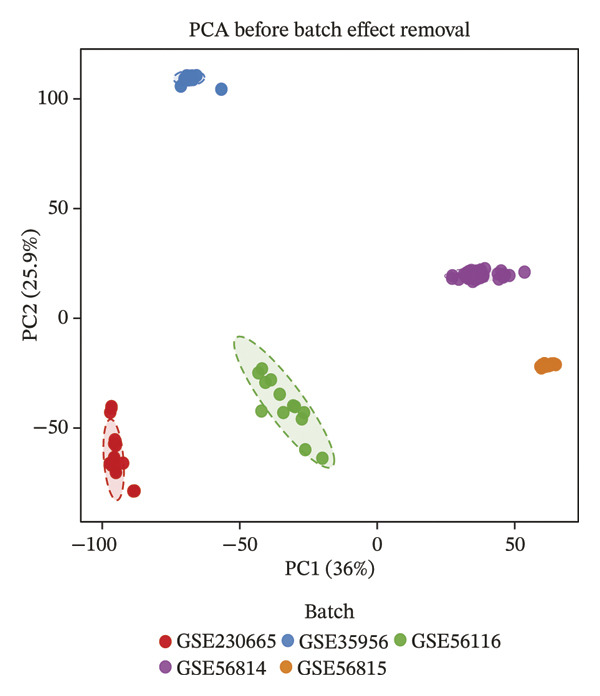
(a)

**Figure:**
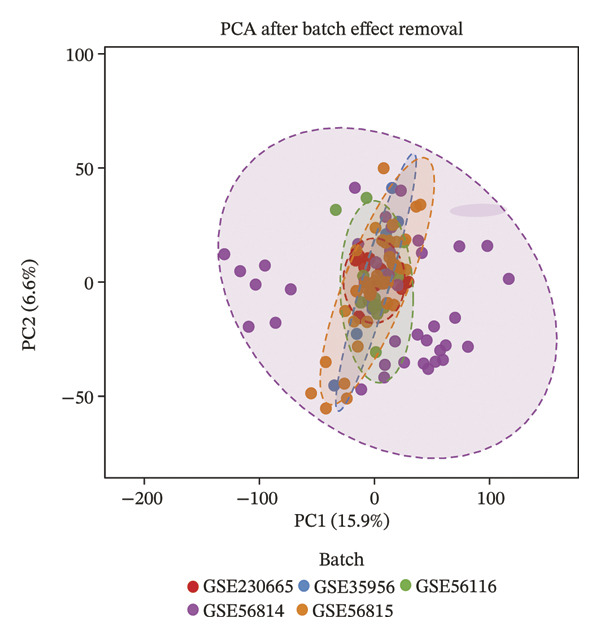
(b)

**Figure:**
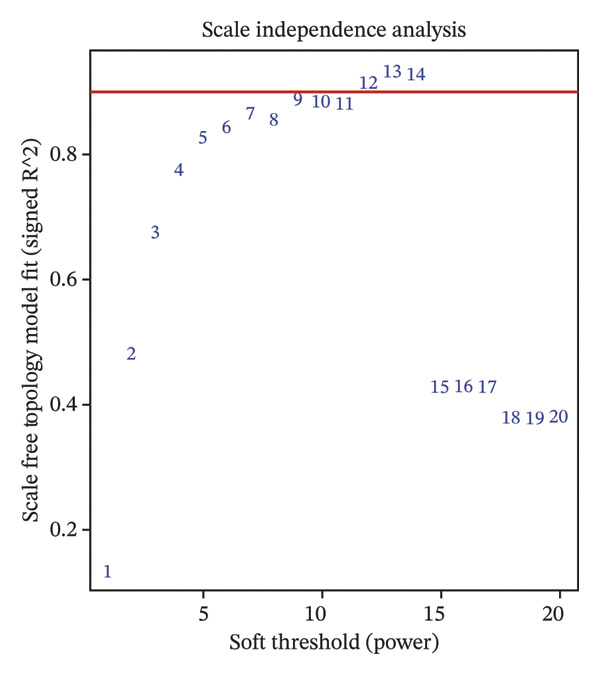
(c)

**Figure:**
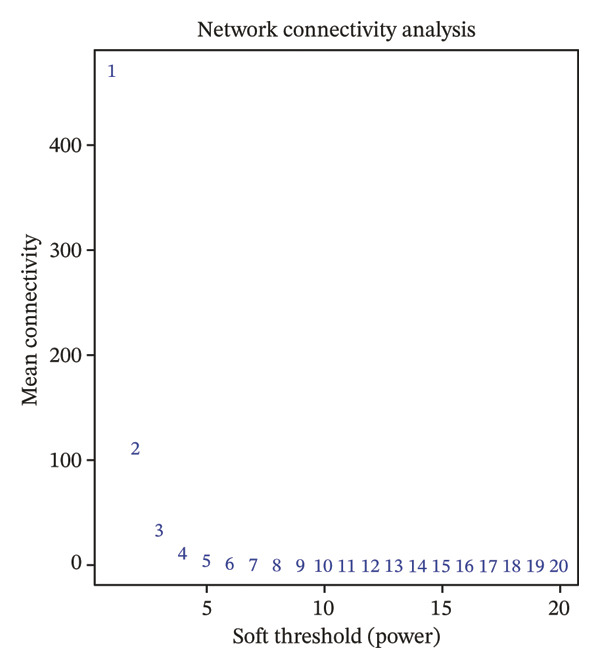
(d)

**Figure:**
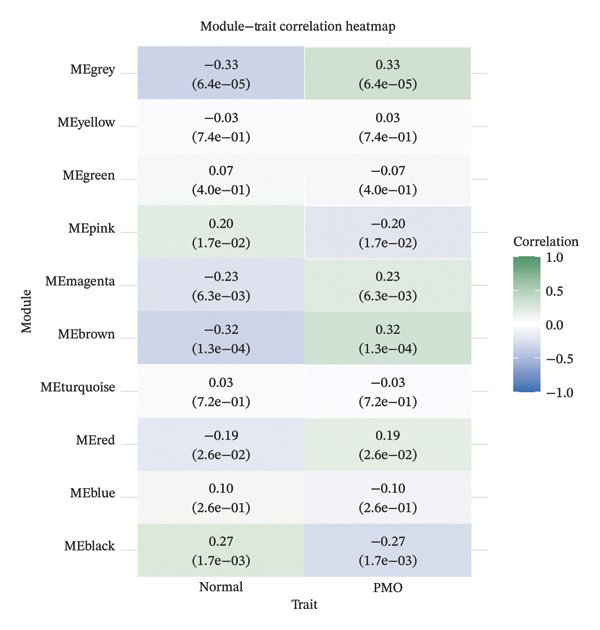
(e)

### 3.3. Identification of Key Predictive Genes for OP Through Machine Learning and SHAP Analysis

Comprehensive machine learning modeling encompassing 108 algorithms was performed on the 64 intersecting genes. We found that the RRF model demonstrated favorable performance while demonstrating the lowest complexity and requiring the fewest feature genes (Figures 3(a), 3(b), 3(c)); thus, it was selected as the representative predictive model.

FIGURE 3Machine learning model construction and SHAP‐based interpretable analysis for identifying key predictive genes in osteoporosis. (a) Venn diagram of shared differentially expressed genes and significant module genes. (b) Performance comparison of 108 machine learning algorithms. (c) Two‐dimensional bubble plot of gene number versus model complexity for algorithms with tied optimal performance. (d) SHAP beeswarm plot of feature genes in the RF model. (e) Feature cumulative contribution curve. Note: The SHAP analysis provides insights into the predictive importance and contribution patterns of features within the model, illustrating associations rather than direct biological causality. Statistical significance was determined where applicable. ^∗^ *p* < 0.05, ^∗∗^ *p* < 0.01, ^∗∗∗^ *p* < 0.001, and ns, not significant.

**Figure:**
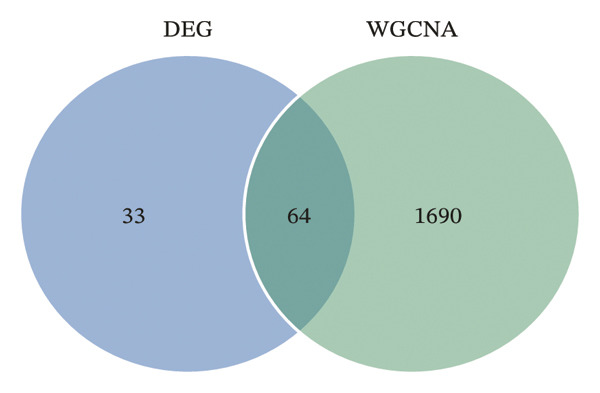
(a)

**Figure:**
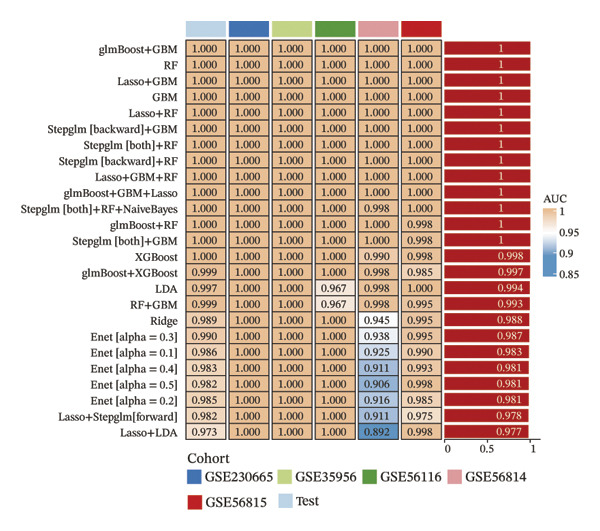
(b)

**Figure:**
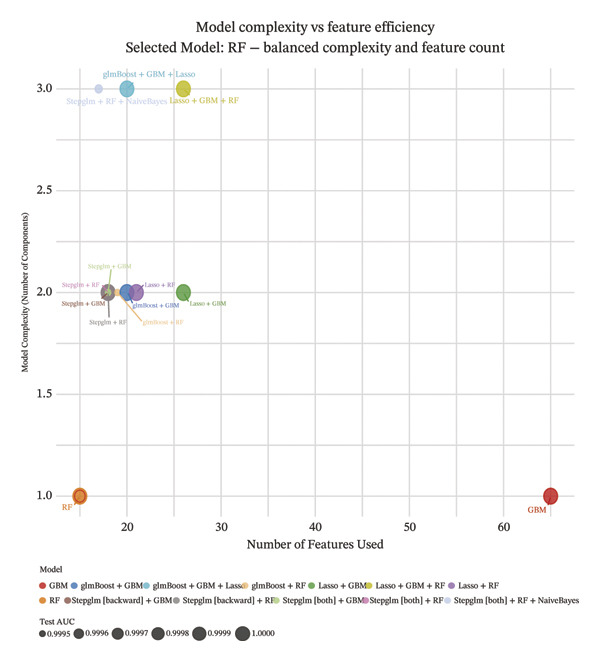
(c)

**Figure:**
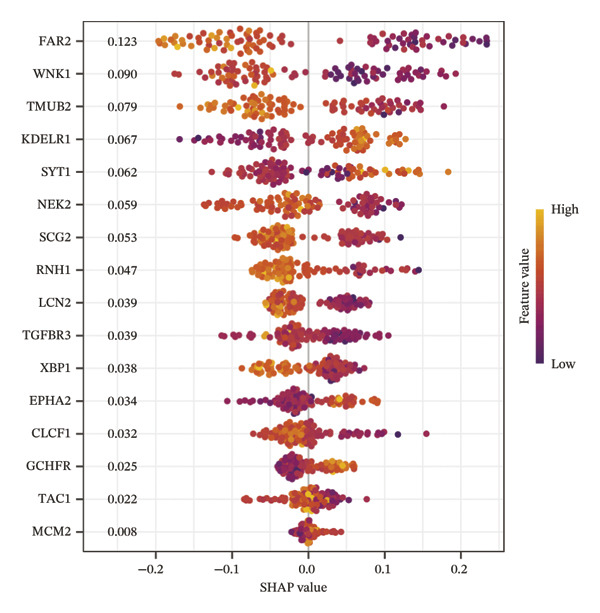
(d)

**Figure:**
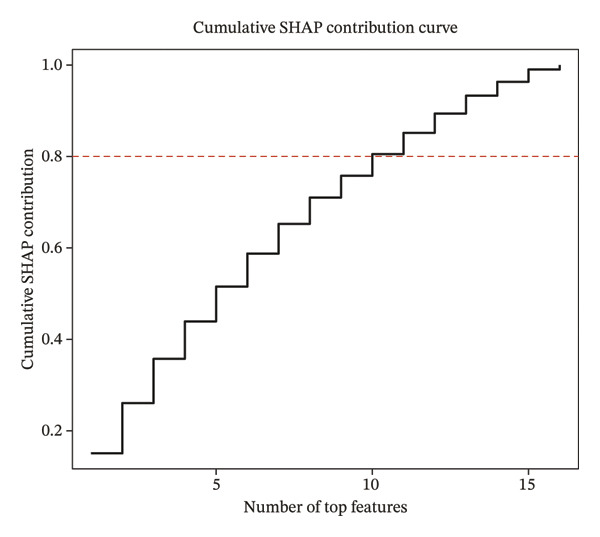
(e)

Through in‐depth interpretation using SHAP analysis, we gained detailed insights into the prediction mechanism of the RF model. Among the 16 feature genes, FAR2 exhibited the highest SHAP importance (0.1228), and its expression level showed a negative correlation with SHAP values. This indicates that increased expression of this gene is associated with a lower model‐predicted probability of disease, suggesting a potential protective role. WNK1 and TMUB2 emerged as the subsequent most influential predictors following FAR2, with SHAP importance values of 0.090 and 0.079, respectively. Both genes demonstrated negative influence patterns consistent with the primary protective factor (Figure 3(d)). Notably, the top 10 feature genes collectively contributed up to 80.5% to the model’s predictions, indicating they constitute the core gene set driving PMO risk prediction (Figure 3(e)).

### 3.4. MR Analysis Reveals a Genetically Predicted Association Between TGFBR3 and OP Risk

To further explore the potential causal relationships between feature genes and OP at the genetic level, we performed a two‐sample MR analysis. eQTLs of the top 10 feature genes were used as exposures, and the OP phenotype from the FinnGen database (finngen_R12_M13_OSTEOPOROSIS) served as the outcome. Results from the IVW method indicated a significant negative genetically predicted association between TGFBR3 and OP risk (OR = 0.675, 95% CI: 0.466–0.977, *p* = 0.037), suggesting that each unit increase in the TGFBR3 expression level was associated with an approximately 32.5% reduction in OP risk. The direction of effect estimates from other MR methods was consistent with the IVW approach although they did not reach statistical significance (Figure 4(a)). Sensitivity analyses (*Q* = 6, *p* = 0.509) further confirmed the robustness of this genetically predicted association, revealing no evidence of significant heterogeneity or pleiotropic bias (Figures 4(b), 4(c), 4(d), and 4(e)). Based on this genetic evidence, TGFBR3 was ultimately selected as the key feature gene for subsequent functional mechanistic investigations.

FIGURE 4Assessment of causal relationships between key feature genes and osteoporosis using two‐sample Mendelian randomization. (a) Scatter plot of MR results. (b) Forest plot displaying individual causal effects of each instrumental variable. (c) Funnel plot for visualizing potential directional pleiotropy. (d) Leave‐one‐out sensitivity analysis plot showing the impact of sequentially removing each instrumental variable on the overall causal estimate. (e) SNP effect distribution plot for detecting horizontal pleiotropy.

**Figure:**
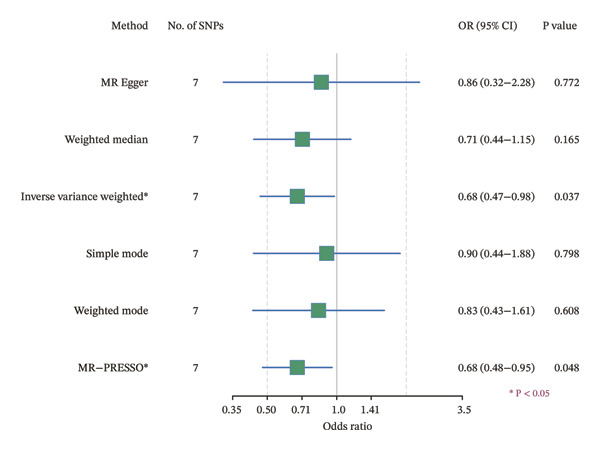
(a)

**Figure:**
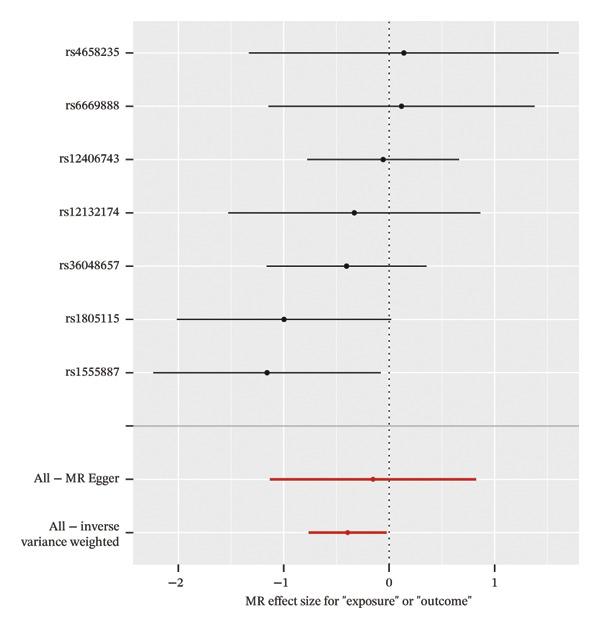
(b)

**Figure:**
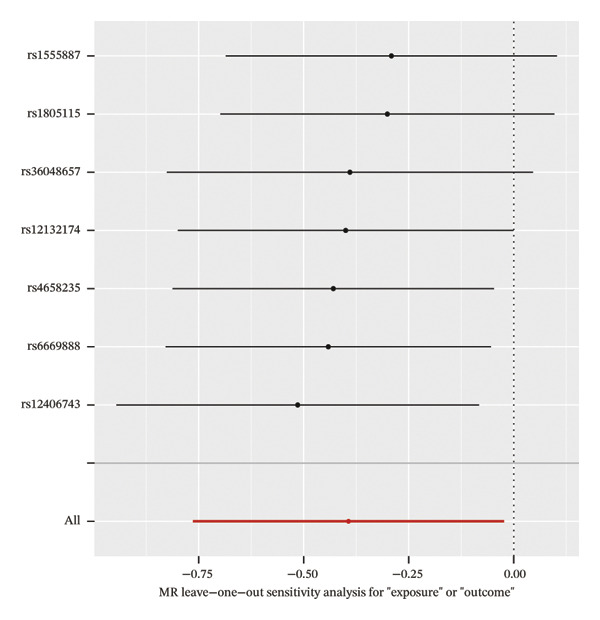
(c)

**Figure:**
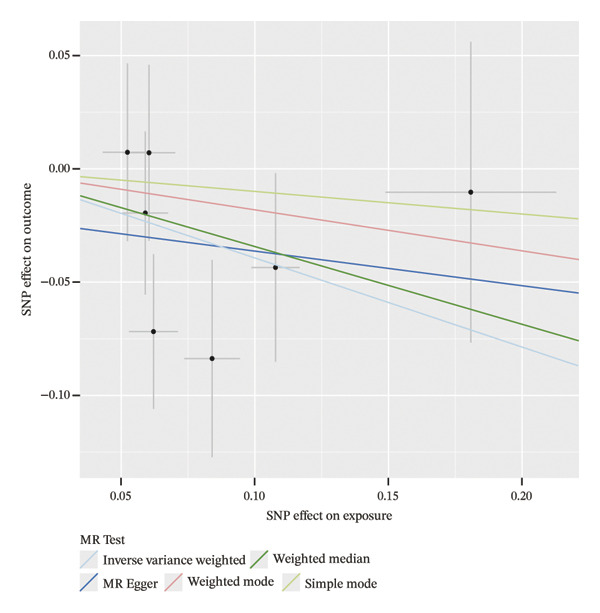
(d)

**Figure:**
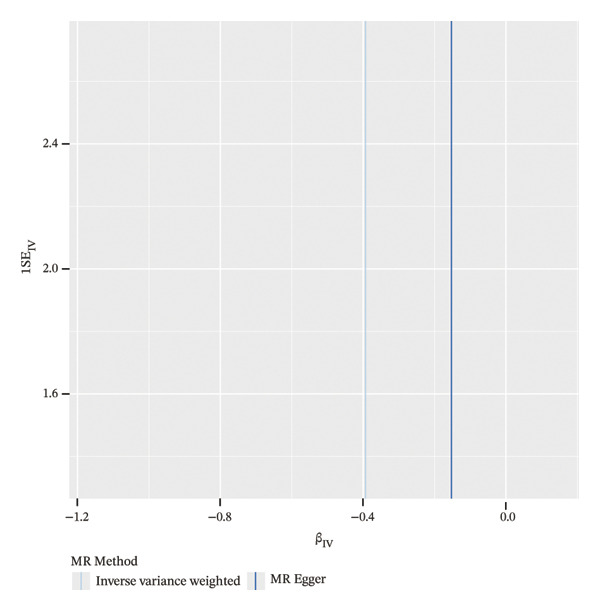
(e)

### 3.5. Validation of Bone Quality and Mechanical Properties in Animal Models

Micro‐CT was used to scan femurs from the model and control group mice, obtaining three‐dimensional reconstructed images and two‐dimensional cross‐sectional images (Figure 5(a)). Bone microstructural parameters were collected from standardized regions of interest and analyzed for differences. Micro‐CT scanning revealed a significant decrease in the overall bone mass in the model group: Bone mineral density, bone volume fraction, Ct.Th, Tb.N, and Tb.Th were all significantly reduced compared to the control group, while the structure model index was significantly increased; although Ct.Ar/Tt.Ar and Tb.Sp showed decreasing trends, these changes did not reach statistical significance (Figure 5(b)). In conclusion, the micro‐CT results showed bone loss and impaired bone microstructure in the OVX group.

FIGURE 5Bone microstructure and mechanical properties in osteoporotic mice. (a) Three‐dimensional reconstructed micro‐CT images of femurs from control and OVX groups. (b) Comparative analysis of bone structural indices. (c) Comparative analysis of bone mechanical indices. Data are presented as mean ± SD. Statistical significance was determined by Student’s *t* test or one‐way ANOVA. ^∗^ *p* < 0.05, ^∗∗^ *p* < 0.01, ^∗∗∗^ *p* < 0.001, and ns, not significant.

**Figure:**
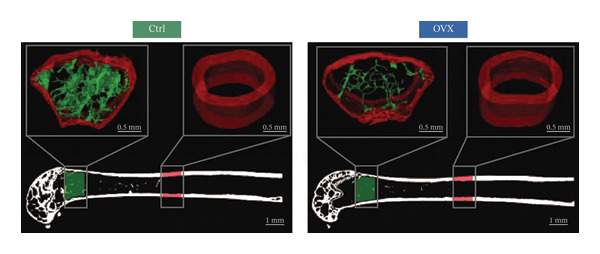
(a)

**Figure:**
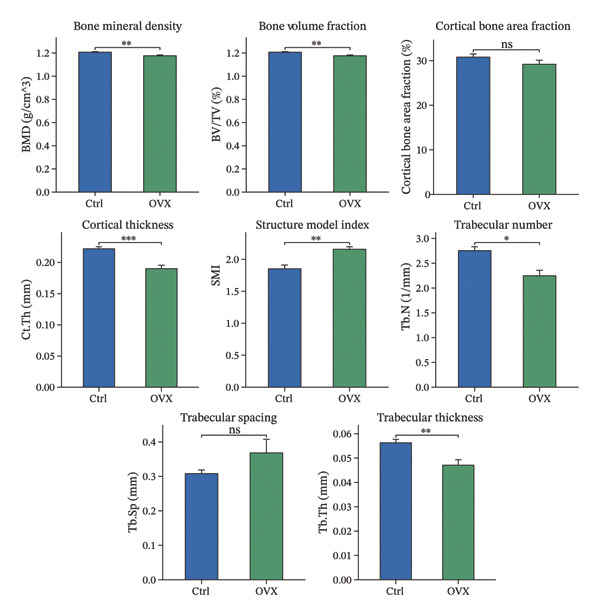
(b)

**Figure:**
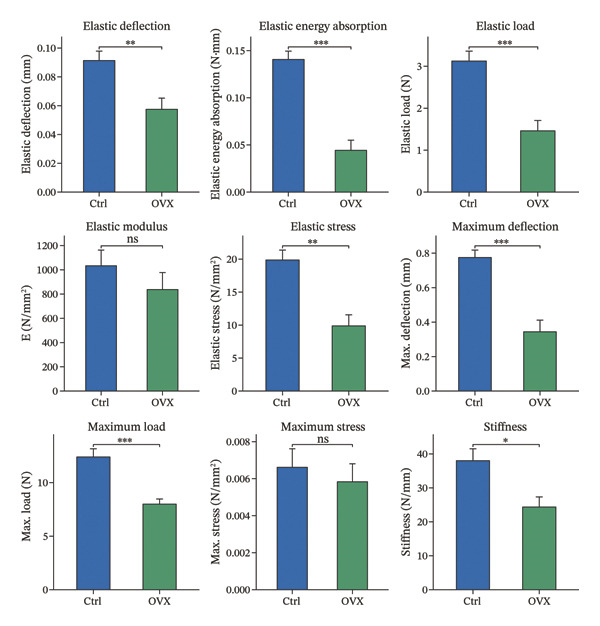
(c)

In addition to the microstructural analysis, three‐point bending tests were conducted to investigate bone mechanical competence. The OVX group demonstrated significant impairment in the biomechanical properties, as demonstrated by the significant decreases in the maximum load, stiffness, elastic load, elastic energy absorption, and related displacement parameters. The maximum stress presented a downward trend, but without statistical significance (Figure 5(c)). Based on the reported bone microarchitecture damage and functional defects, we confirmed that a valid osteoporotic model was successfully established and mimics the typical postmenopausal bone fragility.

### 3.6. Experimental Validation of TGFBR3 Downregulation in PMO Models

Western blot and qRT‐PCR results revealed that expression levels of TGFBR3, OPG, and OCN were markedly downregulated, while the expression level of RANKL was markedly upregulated in the model group. The reliability of the model was further verified, and the expression level of TGFBR3 was downregulated in the process of OP (Figure 6).

FIGURE 6Validation of TGFBR3 and related bone metabolism protein and mRNA expression. (a) Representative western blot bands and corresponding quantitative histogram of relative protein expression. (b) Histogram of relative protein expression levels. (c) Histogram of relative mRNA expression levels. Data are presented as mean ± SD. Statistical significance was determined by Student’s *t* test or one‐way ANOVA. ^∗^ *p* < 0.05, ^∗∗^ *p* < 0.01, ^∗∗∗^ *p* < 0.001, and ns, not significant.

**Figure:**
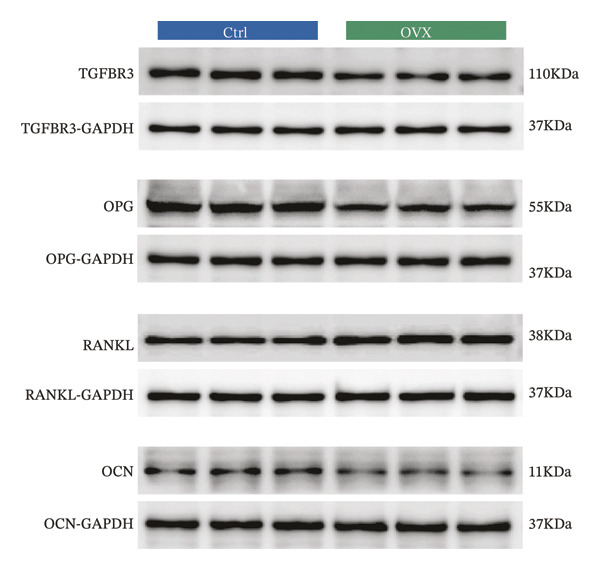
(a)

**Figure:**
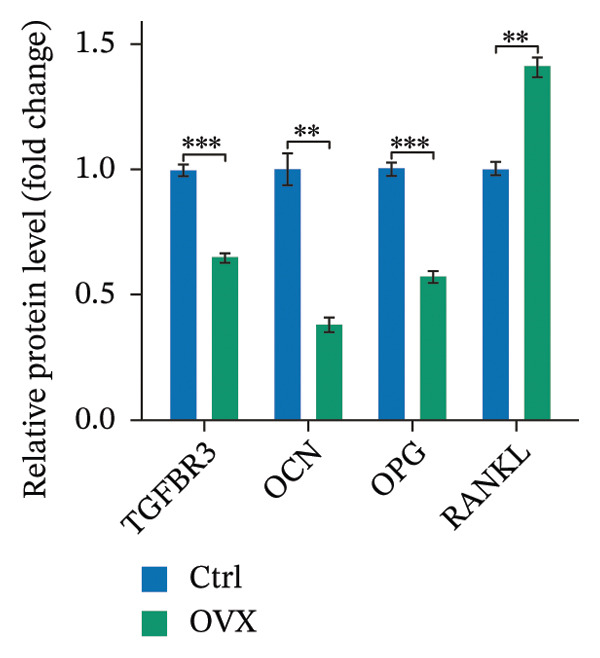
(b)

**Figure:**
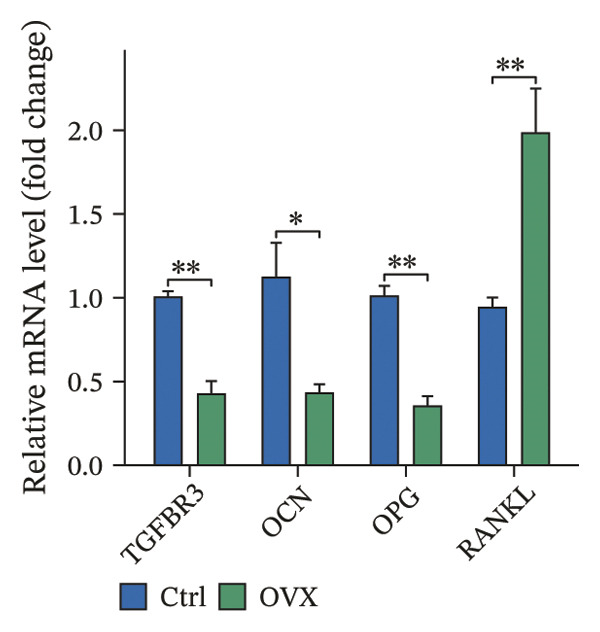
(c)

### 3.7. Experimental Verification of Downregulation of TGFBR3 in PMO Models

To evaluate the biological significance of TGFBR3, we performed comprehensive correlation analyses between TGFBR3 levels and multidimensional bone metabolic markers. Initially, at the transcriptional level, Pearson correlation analysis revealed that TGFBR3 mRNA expression was significantly and positively correlated with osteogenic markers, including OCN and OPG, while showing a significant negative correlation with the osteoclastic marker RANKL (Figure 7(a)). Consistent with these findings, protein‐level analysis confirmed that TGFBR3 was significantly and positively correlated with OCN, while showing a significant negative correlation with RANKL (Figure 7(b)). Regarding OPG protein levels, although a positive correlation trend was observed, it did not reach statistical significance (Figure 7(b)). We further explored the relationship between TGFBR3 and bone microarchitecture through Micro‐CT parameters. Transcriptional TGFBR3 mRNA showed significant positive correlations with BMD, BV/TV, Tb.Th, and Ct.Th and a significant negative correlation with SMI (Figure 7(c)). Positive trends were observed for Tb.N and Ct.Ar/Tt.Ar and a negative trend for Tb.Sp, though these did not reach statistical significance (Figure 7(c)). At the protein level, TGFBR3 expression was significantly and positively correlated with BMD, BV/TV, Tb.Th, Ct.Ar/Tt.Ar, and Ct.Th, while remaining significantly and negatively correlated with SMI (Figure 7(d)). Trends for Tb.N and Tb.Sp remained consistent with the mRNA results but were nonsignificant (Figure 7(d)).

FIGURE 7Correlation analyses of TGFBR3 with bone metabolic markers, microstructural parameters, and mechanical properties. (a) Correlation analysis between TGFBR3 and bone metabolism markers at the protein level. (b) Correlation analysis between TGFBR3 and bone metabolism markers at the mRNA level. (c) Correlation analysis between micro‐CT parameters and relative TGFBR3 mRNA expression. (d) Correlation analysis between micro‐CT parameters and relative TGFBR3 protein expression. (e) Correlation analysis between mechanical parameters and relative TGFBR3 mRNA expression. (f) Correlation analysis between mechanical parameters and relative TGFBR3 protein expression. Note: Statistical significance was determined by Pearson correlation analysis. Significant correlations are denoted as ^∗^ *p* < 0.05, ^∗∗^ *p* < 0.01, and ^∗∗∗^ *p* < 0.001. Comparisons without stars represent those that are not statistically significant (ns).

**Figure:**
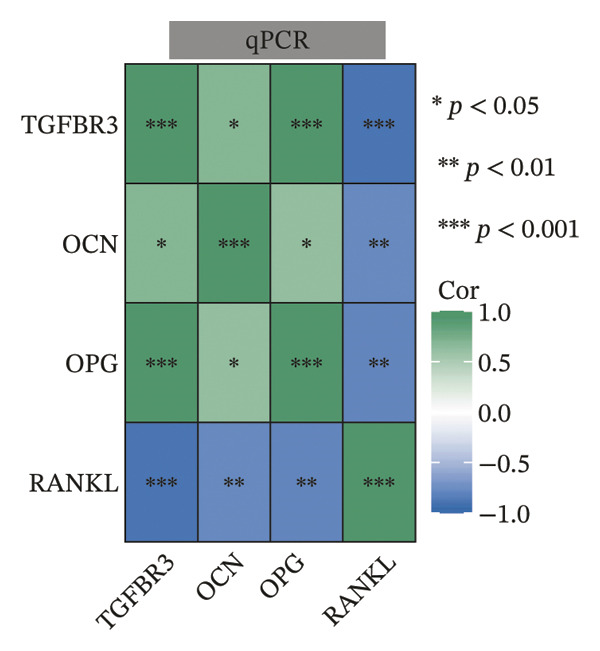
(a)

**Figure:**
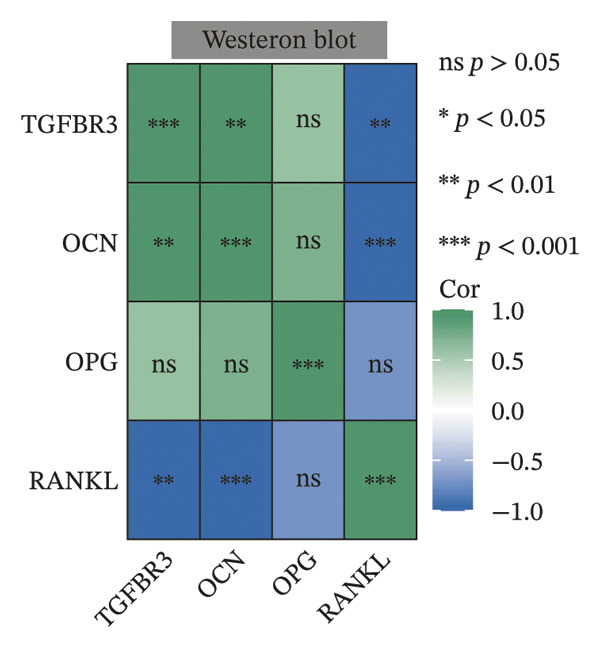
(b)

**Figure:**
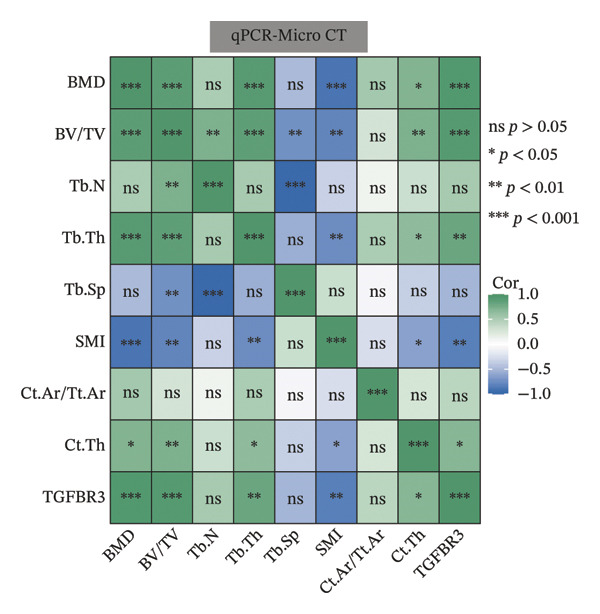
(c)

**Figure:**
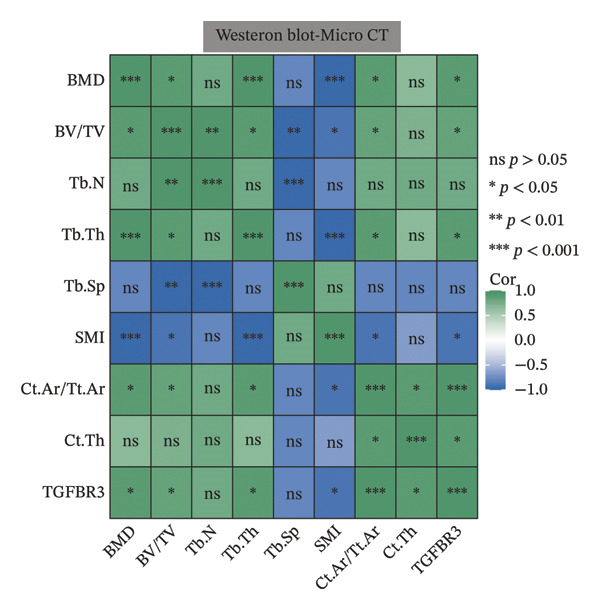
(d)

**Figure:**
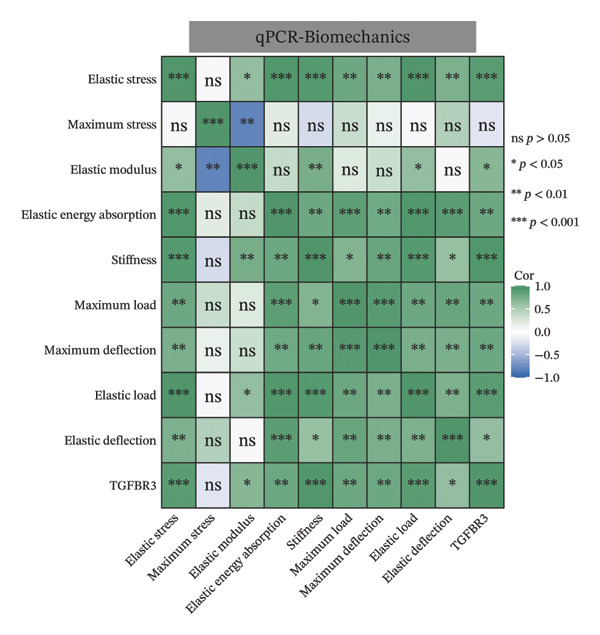
(e)

**Figure:**
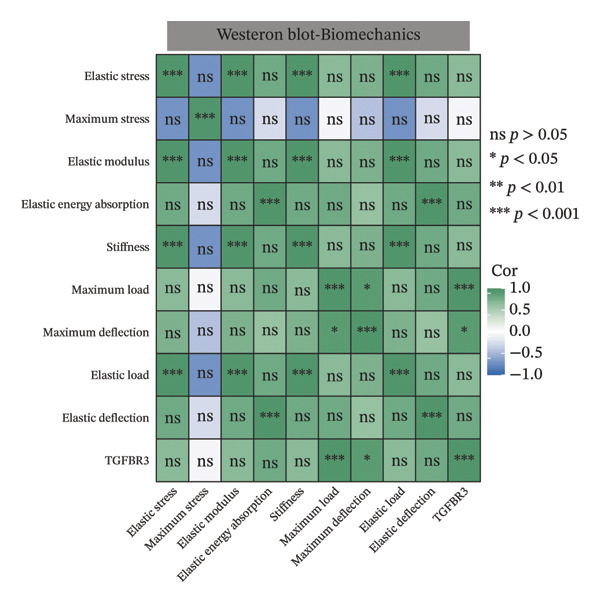
(f)

Furthermore, biomechanical analysis demonstrated that TGFBR3 expression closely correlated with bone mechanical competence. Transcriptionally, TGFBR3 mRNA was significantly and positively correlated with eight critical mechanical parameters: elastic stress, elastic modulus, elastic energy absorption, stiffness, maximum load, maximum deflection, elastic load, and elastic deflection (Figure 7(e)). Maximum stress showed a weak positive trend without reaching statistical significance (Figure 7(e)). At the protein level, the overall correlation trends were consistent with the mRNA findings; however, statistically significant positive correlations with TGFBR3 protein expression were maintained only for maximum load and maximum deflection (Figure 7(f)). In summary, our multidimensional evidence—spanning from transcriptional control and metabolic markers to the maintenance of microarchitecture and skeletal mechanics—implicates TGFBR3 as a vital coordinator of bone metabolic equilibrium and skeletal competence (Figure 7).

### 3.8. Single‐Cell Transcriptomic Profiling Reveals Cell‐Type‐Specific Expression of TGFBR3 in the Bone Marrow Microenvironment and Its Downregulation in OP

To resolve TGFBR3 functions at single‐cell resolution, we analyzed integrated human bone marrow transcriptomic datasets. TGFBR3 was found to be significantly enriched in key cellular populations, such as mesenchymal stem cells, vascular endothelial cells, and T cells (Figures 8(a), 8(b), 8(c)). The expression of TGFBR3 in the independent CD271^+^bone marrow mesenchymal stem cell dataset was confirmed (Figures 8(d), 8(e), and 8(f)), suggesting its involvement in stromal maintenance, vascular function, and immune modulation.

FIGURE 8Single‐cell transcriptomic profiling reveals cell‐type‐specific expression of TGFBR3 in the bone marrow microenvironment. (a) UMAP plot showing annotated cell types in the normal bone marrow microenvironment. (b) Expression localization of TGFBR3 in human bone marrow single cells. (c) Bar plot showing TGFBR3 expression across normal bone marrow cell subsets. (d) UMAP plot showing cell type annotations in the Wang dataset (CD271^+^ MSCs). (e) UMAP visualization of TGFBR3 expression in CD271^+^ MSC subsets. (f) Bar plot showing TGFBR3 expression in CD271^+^ MSC subsets. (g) Bar plot comparing TGFBR3 expression between osteoporosis and control groups within specific cell subsets.

**Figure:**
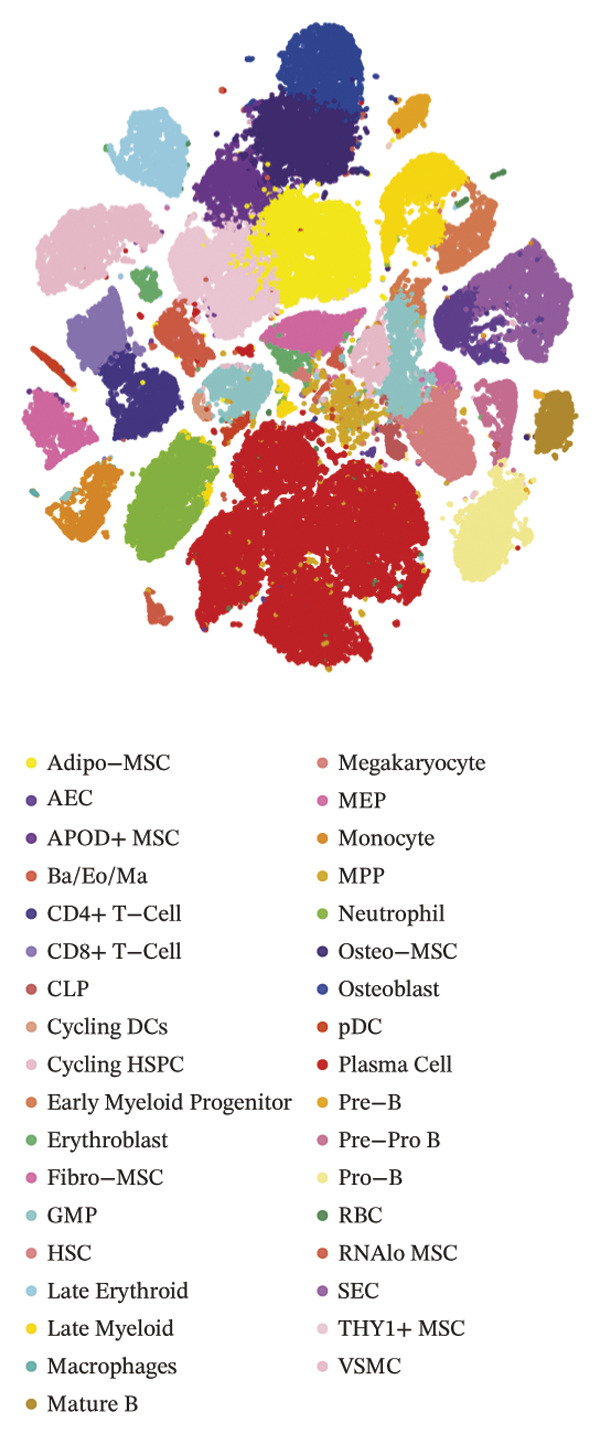
(a)

**Figure:**
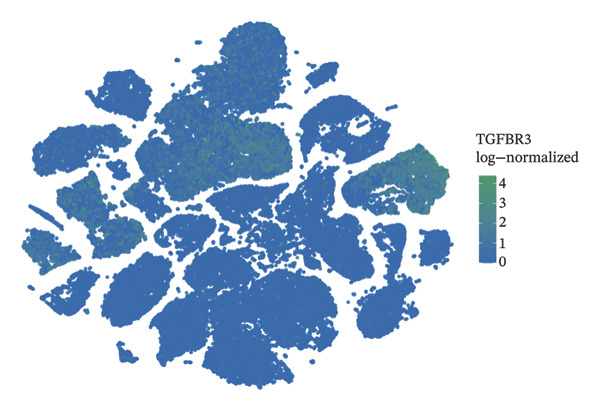
(b)

**Figure:**
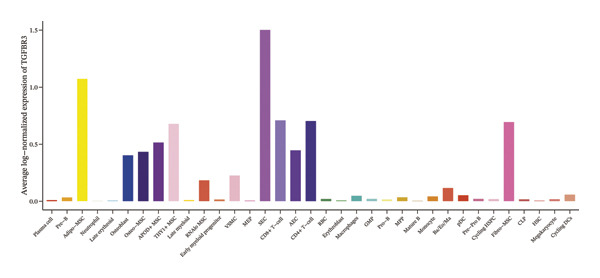
(c)

**Figure:**
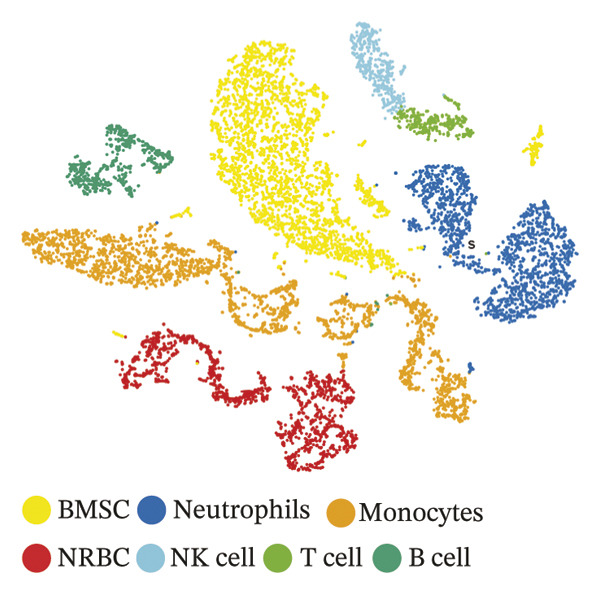
(d)

**Figure:**
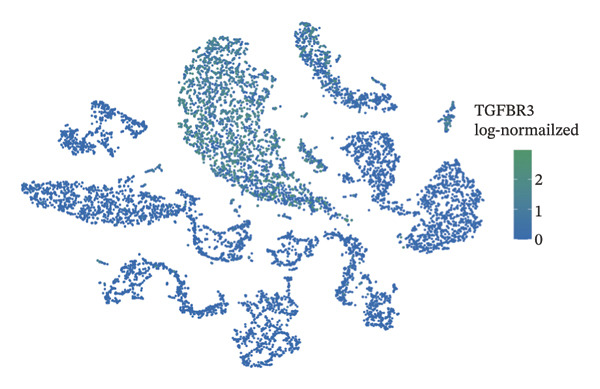
(e)

**Figure:**
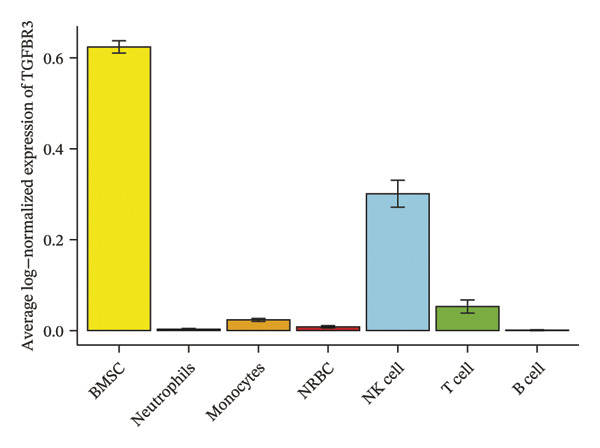
(f)

**Figure:**
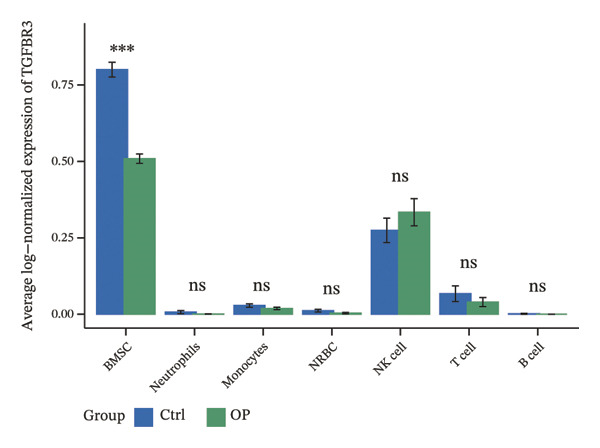
(g)

Building on these cellular localization findings, we compared TGFBR3 expression between OP patients and controls. Osteoporotic bone marrow mesenchymal stem cells exhibited significantly reduced TGFBR3 levels (Figure 8(g)), supporting their hypothesized protective function in bone metabolism.

### 3.9. Functional Enrichment Suggests TGFBR3 Exerts Bone‐Protective Effects Through a Multilevel Network

Based on transcriptomic data from bone and bone marrow tissues, we systematically identified six key TGFBR3‐associated pathway intersection sets by integrating GSVA and GSEA enrichment analyses, thereby revealing the multilevel regulatory network of TGFBR3 as a bone mass protective factor in PMO (Figure 9(a)). These six sets specifically include (1) the bone tissue consistently upregulated pathway set (intersection of TGFBR3_Bone_GSVA_Up and TGFBR3_Bone_GSEA_Up, containing 23 pathways), (2) the bone tissue consistently downregulated pathway set (intersection of TGFBR3_Bone_GSVA_Down and TGFBR3_Bone_GSEA_Down, containing 2 pathways), (3) the bone marrow–bone tissue reverse regulation pathway set (all methods) (intersection of TGFBR3_Bone_GSEA_Down, TGFBR3_Bone_GSVA_Down, TGFBR3_Marrow_GSEA_Up, and TGFBR3_Marrow_GSVA_Up, containing 2 pathways), (4) the bone marrow–bone tissue reverse regulation pathway set (GSVA) (intersection of TGFBR3_Bone_GSEA_Down, TGFBR3_Bone_GSVA_Down, and TGFBR3_Marrow_GSVA_Up, containing 2 pathways), (5) the putative cross‐tissue regulation pathway set (intersection of TGFBR3_Marrow_GSVA_Up, TGFBR3_Bone_GSEA_Down, and TGFBR3_Marrow_GSEA_Up, containing 1 pathway), and (6) the cross‐tissue consistently upregulated pathway set (intersection of TGFBR3_Marrow_GSVA_Up, TGFBR3_Bone_GSVA_Up, and TGFBR3_Bone_GSEA_Up, containing 1 pathway) (Figure 9(b)).

FIGURE 9Functional enrichment analysis exploring the TGFBR3 regulatory network reveals its bone protection mechanism in osteoporosis. (a) UpSet plot of functional enrichment analysis for bone and bone marrow tissues. (b) Detailed pathway intersections identified in the TGFBR3 analysis.

**Figure:**
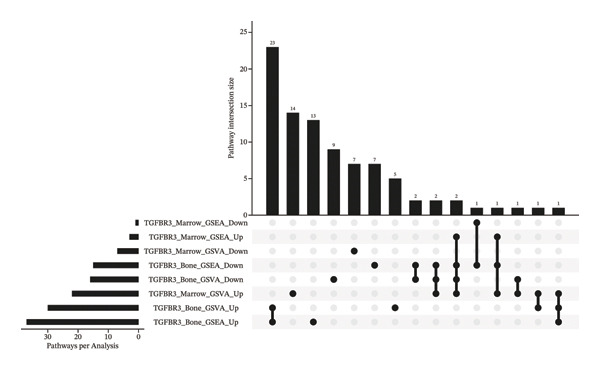
(a)

**Figure:**
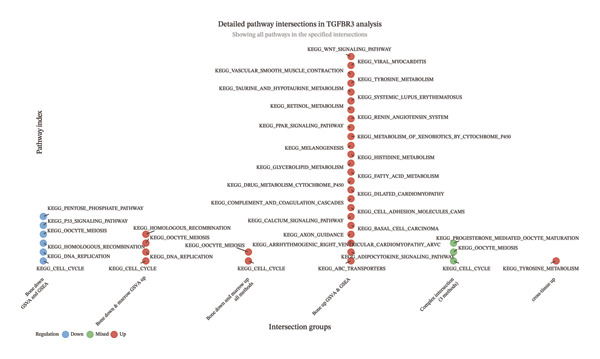
(b)

These sets can be categorized into three functional modules: intrabone osteogenic regulation, bone marrow–bone tissue signaling crosstalk, and cross‐tissue coordination mechanisms. Within bone tissue, we identified two highly consistent pathway sets: the “bone tissue consistently upregulated” set was enriched in classical pro‐osteogenic pathways such as Wnt, TGF‐β, and calcium signaling, indicating that TGFBR3 is a key positive regulator of the osteogenic differentiation program in bone tissue, whereas the “bone tissue consistently downregulated” set contained cell cycle and DNA replication pathways, revealing an important role for TGFBR3 in maintaining osteoblast proliferative capacity. At the intertissue communication level, the two “bone marrow–bone tissue reverse regulation” sets exhibited opposite regulatory patterns, suggesting that TGFBR3 plays a crucial role in coordinating bone marrow microenvironment and bone tissue function, and its downregulation leads to dysregulation of cross‐tissue signaling. Furthermore, the “cross‐tissue consistently upregulated” pathway implies that TGFBR3 may be involved in synergistic protective effects across multiple tissues.

### 3.10. Immune Cell Infiltration Characteristics of TGFBR3 in PMO

In the bone tissue microenvironment, TGFBR3 exhibited immunomodulatory capacity, significantly positively correlated with stromal score (*r* = 0.965), myeloid lineages (dendritic cells, *r* = 0.916; macrophages, M1: *r* = 0.937; M2: *r* = 0.903), and cytotoxic populations (lymphocytes, *r* = 0.916; NK cells, *r* = 0.881). More interestingly, the polarization pattern of T cell subsets was remarkably distinct: robust positive correlations with *T* follicular helper cells (*r* = 0.916), γδ T cells (*r* = 0.915), and Th1 cells (*r* = 0.860), while strongly negative correlation with Th2 cells (*r* = −0.909). Therefore, our results indicated that TGFBR3 was critically involved in maintaining osteoimmune balance by regulating Th1/Th2 balance regulation.

In contrast, the positive correlation pattern of TGFBR3 in bone marrow tissue was much weaker: significant positive correlations with adaptive immune components [cytotoxic lymphocytes, *r* = 0.484; CD8+ T cells, *r* = 0.302–0.462; B cells, *r* = 0.265–0.331], while significantly negative correlations with innate immune components [activated mast cells, *r* = −0.310], as well as monocytes [*r* = −0.303]. These distinct patterns of tissue‐specific regulation provide insights into the complex involvement of TGFBR3 in both adaptive and innate immune pathways.

**FIGURE 10:**
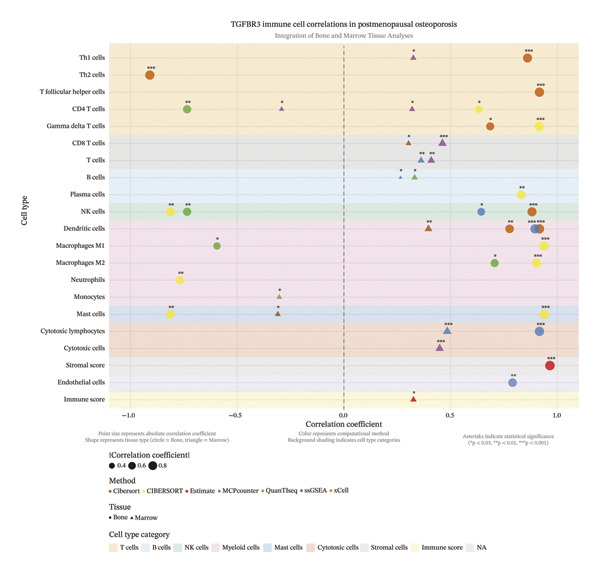
Comprehensive analysis of immune cell infiltration associated with TGFBR3 in postmenopausal osteoporosis. Statistical significance of correlations was determined by Pearson correlation analysis. ^∗^ *p* < 0.05, ^∗∗^ *p* < 0.01, ^∗∗∗^ *p* < 0.001, and ns, not significant.

A comprehensive comparative analysis between different tissue compartments not only identified the basal property of the target gene immunomodulatory network but also validated single‐cell transcriptomic results. The specific enrichment of TGFBR3 in T cells and DCs identified at a single cell level was further confirmed by stable positive correlations identified in bulk tissue, which defined its core immunomodulatory network in osteoblasts and bone marrow. These convergent results further improved our understanding from simple cellular localization to functional tissue‐level correlation. In addition, the specific enrichment of B cell and macrophage correlation patterns in different tissue compartments further highlight the microenvironment‐dependent specificity of TGFBR3 function (Figure 10).

## 4. Discussion

PMO pathogenesis involves dysregulated interactions among bone tissue, marrow microenvironment, and systemic immunity—components traditionally investigated separately. To bridge this gap, we applied an integrated analytical strategy integrating cross‐tissue transcriptomics with causal inference to identify TGFBR3 as a novel osteoprotective factor and delineate its multilevel regulatory network in bone metabolism.

### 4.1. Cross‐Tissue Analytical Paradigm: Extracting the Core From Noise

We found a conserved transcriptional program where 97 dysregulated genes were consistently modulated across bone, marrow, and blood compartments. This molecular signature represents coordinated transcriptional dysregulation among bone, marrow, and blood compartments due to estrogen deficiency. Upon this foundation, we applied a stepwise refinement strategy: Following feature refinement to 64 genes via WGCNA, converging evidence from machine learning, SHAP analysis, and MR pinpointed TGFBR3 as the pivotal regulator. This two‐tiered filtering strategy—from bulk transcriptome screening to network‐based filtering and predictive modeling of core genes—successfully distinguished transcriptional regulation at the system level from tissue background variation.

### 4.2. Osteoimmune–Osteogenic Integration Through Cellular Specialization

Single‐cell transcriptomics revealed TGFBR3’s cellular expression pattern, localized primarily in two functionally complementary populations within bone marrow: two types of mesenchymal stem cells and two types of immune cells (T cells and dendritic cells). This cellular localization suggests that TGFBR3 regulates bone formation and immune response. Specifically, the expression of TGFBR3 in mesenchymal stem cells positively correlated with the activation of osteogenic pathway (Wnt/β‐catenin and TGF‐β signaling) supported its role in bone formation [44], while in immune compartments, positive correlations with pro‐osteogenic populations (Th1 cells and cytotoxic lymphocytes) and negative correlations with Th2 cells suggest its role in establishing an immune microenvironment conducive to bone formation [6, 45, 46].

### 4.3. Multilevel Regulation of Skeletal Homeostasis

Based on the multidimensional evidence above, we propose that TGFBR3 maintains skeletal homeostasis through a sophisticated multilevel regulatory network (Figure 6). Under physiological conditions, this regulatory network acts through a three‐tiered cooperative mechanism at the molecular level, and TGFBR3 directly promotes osteogenic differentiation by modulating the activities of Wnt/β‐catenin and TGF‐β signaling pathways [44, 47]; at the cellular level, it establishes an immune microenvironment conducive to bone formation by modulating the Th1/Th2 balance [6, 45, 46]; at the tissue level, it mediates signaling crosstalk between bone marrow and bone tissue. This “molecule–cell–tissue” multilevel regulatory network allows for fine‐grained control of skeletal homeostasis [48].

Therefore, we conclude that in the pathologic state of PMO, estrogen deficiency‐induced TGFBR3 downregulation disrupts cooperative network cascading failure at multiple levels from the blockade of molecular pathways and disruption of cellular functions to the loss of intertissue signaling coordination, promoting the development of PMO.

### 4.4. Limitations and Future Work

There are several limitations. First, heterogeneity in sample sizes, population composition, and technical platforms in publicly available repositories may lead to great variability in result patterns of transcriptomic data [49]. Furthermore, although the initial identification of tissue‐specific dysregulation relied on representative high‐quality datasets (such as GSE230665 for femoral tissue and GSE35956 for bone marrow), the relatively limited sample sizes and platform‐specific variations inherent in individual public datasets represent a potential constraint. This underscores the necessity of our integrated multisource analytical paradigm; by bridging disparate transcriptomic profiles from bone, marrow, and blood, we were able to filter out tissue‐specific noise and extract conserved molecular signatures of PMO at a systemic level, thereby enhancing the robustness and generalizability of our findings.

Second, MR analyses were mainly based on European population eQTL data and need to be validated in ethnic populations [16]. Although our MR results support a causal role for TGFBR3 in OP, this conclusion hinges on the three core IV assumptions. Despite detailed sensitivity analyses showing no evidence of directional pleiotropy, we cannot entirely rule out residual pleiotropy from unmeasured variables. Furthermore, while the observed cross‐tissue conservation of TGFBR3 alleviates concerns, the use of blood‐based eQTLs as proxies for bone tissue expression remains a potential source of bias.

Third, regarding the experimental validation, the OVX mouse model, while the gold standard, represents an acute withdrawal of estrogen. This does not fully recapitulate the gradual physiological transition of human menopause. Additionally, while our animal study confirmed the downregulation of TGFBR3 and its correlation with bone loss, the relatively small sample size (*n* = 6 per group) may limit the precision of the descriptive statistics and the generalizability of the findings to more complex clinical phenotypes.

Finally, the specific cell‐type‐specific molecular actions within the TGFBR3 regulatory network remain to be fully elucidated. Future work should focus on gain‐ and loss‐of‐function studies (e.g., osteoblast‐specific Tgfbr3 knockout mice) to rigorously test the mechanistic validity of this signaling axis and its potential as a precise therapeutic target for PMO.

## 5. Conclusion

In conclusion, our study integrated cross‐tissue multiomics analysis with in vivo experimental validation to identify TGFBR3 as a novel and systemic protective factor in PMO. The significant downregulation of TGFBR3 in both bone tissues and peripheral blood of OVX mice validates our initial computational hypothesis and aligns with current theories emphasizing the pivotal role of TGF‐beta signaling in maintaining bone homeostasis. By demonstrating that TGFBR3 expression correlates with bone mineral density and mechanical integrity across different biological compartments, this research provides strong evidence for TGFBR3 as a potential cross‐tissue biomarker and a promising therapeutic target for PMO.

**Nomenclature**
PMO: Postmenopausal osteoporosis
PBMC: Peripheral blood mononuclear cell
WGCNA: Weighted gene coexpression network analysis
PCA: Principal component analysis
DEG: Differentially expressed gene
RF: Random forest
SHAP: SHapley Additive exPlanations
MR: Mendelian randomization
IVW: Inverse variance weighted
SNP: Single‐nucleotide polymorphism
eQTL: Expression of quantitative trait locus
OVX: Ovariectomized
Micro‐CT: Microcomputed tomography
BMD: Bone mineral density
BV/TV: Bone volume fraction
SMI: Structure model index
Tb.N: Trabecular number
Tb.Th: Trabecular thickness
Tb.Sp: Trabecular separation
Ct.Th: Cortical thickness
OPG: Osteoprotegerin
OCN: Osteocalcin
RANKL: Receptor activator of nuclear factor kappa‐B ligand
qRT‐PCR: Quantitative real‐time polymerase chain reaction
WB: Western blot
UMAP: Uniform manifold approximation and projection
MSC: Mesenchymal stem cell
GSVA: Gene set variation analysis
GSEA: Gene set enrichment analysis
ssGSEA: Single‐sample gene set enrichment analysis

## Author Contributions

Yimin Liu participated in all aspects of this study. Chenxu Xie, Kaiwen Yang, and Xiaoli Hou participated in mouse breeding and animal modeling and conducted micro‐CT detection and three‐point bending tests.

Zixuan Liu, Lei Xing, and Yunpeng Hu performed qRT‐PCR and western blot analysis.

Runtong Liu, Jingyuan Gao, and Qiangqiang Lian carried out statistical data analysis and figure preparation.

Faming Tian, Liu Zhang, and Yongheng Wang provided research guidance, manuscript review, and funding support.

## Funding

This study was supported by the Central Government‐guided Local Science and Technology Development Foundation of Hebei Province (Grant Nos. 226Z7709G and 246Z7744G) and the Basic Scientific Research Foundation of Universities in Hebei Province (Grant No. JYG2021005).

## Ethics Statement

All animal experiments were approved by the Animal Ethics Committee of North China University of Science and Technology and were conducted in accordance with the institutional guidelines.

## Conflicts of Interest

The authors declare no conflicts of interest.

## Supporting Information

Additional supporting information can be found online in the Supporting Information section.

## Supporting information


**Supporting Information 1** Supporting File 1: STROBE‐MR checklist of the recommended items to address in reports of Mendelian randomization studies.

**Supporting Information 2** Supporting File 2: Author checklist of the required items to address in compliance with the journal’s submission and formatting guidelines.

## Data Availability

The datasets used and/or analyzed during the current study are available from the corresponding author upon reasonable request.
