# Supplementary material for: A Cross‐Tissue Multiomics Analysis Reveals the Protective Role of TGFBR3 in Postmenopausal Osteoporosis
Source: Int J Genomics. 2026 Apr 6;2026:6364895. doi: 10.1155/ijog/6364895 (PMC13051796; doi:10.1155/ijog/6364895)
Supplement: Supplementary file 1 — Supporting Information 1 Supporting File 1: STROBE‐MR checklist of the recommended items to address in reports of Mendelian randomization studies. [file IJOG-2026-6364895-s002.docx]

**STROBE-MR checklist of recommended items to address in reports of Mendelian randomization studies**^1^ ^2^

| **Item No.** | **Section** | **Checklist item** | **Page No.** | **Relevant text from manuscript** |
| --- | --- | --- | --- | --- |
| 1 | **TITLE and ABSTRACT** | Indicate Mendelian randomization (MR) as the study’s design in the title and/or the abstract if that is a main purpose of the study | 1 | Abstract: "Two-sample Mendelian randomization was used to inform causal gene-disease relationships" |
|  | **INTRODUCTION** |  | 2-3 | Introduction: "Mendelian randomization utilizes genetic instrumental variables to infer causal relationships between genes and diseases, mitigating confounding biases inherent in traditional observational studies to some extent" |
| 2 | **Background** | Explain the scientific background and rationale for the reported study. What is the exposure? Is a potential causal relationship between exposure and outcome plausible? Justify why MR is a helpful method to address the study question | 2-3 | Introduction: "employing machine learning and Mendelian randomization to identify the key causal gene, TGFBR3" |
| 3 | **Objectives** | State specific objectives clearly, including pre-specified causal hypotheses (if any). State that MR is a method that, under specific assumptions, intends to estimate causal effects | 4-5 | 2.5 Mendelian Randomization Data: Detailed description of eQTLGen consortium and FinnGen database sources |
|  | **METHODS** |  |  |  |
| 4 | **Study design and data sources** | Present key elements of the study design early in the article. Consider including a table listing sources of data for all phases of the study. For each data source contributing to the analysis, describe the following: | 4-5 | 2.5 Mendelian Randomization Data: Detailed description of eQTLGen consortium and FinnGen database sources |
|  | a) | Setting: Describe the study design and the underlying population, if possible. Describe the setting, locations, and relevant dates, including periods of recruitment, exposure, follow-up, and data collection, when available. | 4-5 | 2.5: "comprising cis-eQTLs for 16,989 genes derived from 31,684 blood samples of healthy individuals of European ancestry" |
|  | b) | Participants: Give the eligibility criteria, and the sources and methods of selection of participants. Report the sample size, and whether any power or sample size calculations were carried out prior to the main analysis | 4-5 | 2.5: "healthy individuals of European ancestry" and FinnGen R12 osteoporosis phenotype |
|  | c) | Describe measurement, quality control and selection of genetic variants | 5 | 2.6: "SNPs significantly associated with the exposure at P < 5×10⁻⁸... LD clumping... MAF ≥ 0.01... F-statistic < 10 excluded" |
|  | d) | For each exposure, outcome, and other relevant variables, describe methods of assessment and diagnostic criteria for diseases | 4-5 | 2.5: eQTLs as exposure, finngen_R12_M13_OSTEOPOROSIS as outcome |
|  | e) | Provide details of ethics committee approval and participant informed consent, if relevant | 5 | 2.5: "All contributing genome-wide association studies... were conducted in accordance with ethical standards" |
| 5 | **Assumptions** | Explicitly state the three core IV assumptions for the main analysis (relevance, independence and exclusion restriction) as well assumptions for any additional or sensitivity analysis | 5 | 2.6: Explicit statement of relevance, independence, and exclusion restriction assumptions |
| 6 | **Statistical methods: main analysis** | Describe statistical methods and statistics used | 5 | 2.6: Detailed description of IVW method, sensitivity analyses, and statistical packages |
|  | a) | Describe how quantitative variables were handled in the analyses (i.e., scale, units, model) | 5 | 2.6: Results presented as OR with 95% CI |
|  | b) | Describe how genetic variants were handled in the analyses and, if applicable, how their weights were selected | 5 | 2.6: SNP selection criteria and LD clumping procedures |
|  | c) | Describe the MR estimator (e.g. two-stage least squares, Wald ratio) and related statistics. Detail the included covariates and, in case of two-sample MR, whether the same covariate set was used for adjustment in the two samples | 5 | 2.6: "The inverse variance weighted (IVW) method was used as the primary analysis" |
|  | d) | Explain how missing data were addressed |  | Not applicable |
|  | e) | If applicable, indicate how multiple testing was addressed | 5 | 2.6: "we did not perform multiple testing correction on the Mendelian randomization analysis" |
| 7 | **Assessment of assumptions** | Describe any methods or prior knowledge used to assess the assumptions or justify their validity | 5 | 2.6: MR-Egger intercept test, weighted median, MR-PRESSO, Cochran's Q test |
| 8 | **Sensitivity analyses and additional analyses** | Describe any sensitivity analyses or additional analyses performed (e.g. comparison of effect estimates from different approaches, independent replication, bias analytic techniques, validation of instruments, simulations) | 5 | 2.6: MR-Egger, weighted median, MR-PRESSO, leave-one-out analysis |
| 9 | **Software and pre-registration** |  | 5 | 2.6: "R version 4.3.2 using the TwoSampleMR (version 0.5.6) and MR-PRESSO (version 1.0)" |
|  | a) | Name statistical software and package(s), including version and settings used | 5 | 2.6: Specific software versions mentioned |
|  | b) | State whether the study protocol and details were pre-registered (as well as when and where) |  | Not applicable |
|  | **RESULTS** |  |  |  |
| 10 | **Descriptive data** |  | 6 | 3.4: Sample sizes and basic characteristics implied through data sources |
|  | a) | Report the numbers of individuals at each stage of included studies and reasons for exclusion. Consider use of a flow diagram |  | Not applicable for summary-level MR |
|  | b) | Report summary statistics for phenotypic exposure(s), outcome(s), and other relevant variables (e.g. means, SDs, proportions) | 6 | 3.4: OR and 95% CI reported |
|  | c) | If the data sources include meta-analyses of previous studies, provide the assessments of heterogeneity across these studies |  | Not applicable |
|  | d) | For two-sample MR:  i.  Provide justification of the similarity of the genetic variant-exposure associations between the exposure and outcome samples  ii.  Provide information on the number of individuals who overlap between the exposure and outcome studies | 5 | 2.6: Discussion of sample overlap and methodological approaches |
| 11 | **Main results** |  | 6 | 3.4: "TGFBR3 was significantly protective against PMO (IVW OR = 0.675, 95% CI: 0.466-0.977, P = 0.037)" |
|  | a) | Report the associations between genetic variant and exposure, and between genetic variant and outcome, preferably on an interpretable scale | 6 | 3.4: MR results for top 10 feature genes |
|  | b) | Report MR estimates of the relationship between exposure and outcome, and the measures of uncertainty from the MR analysis, on an interpretable scale, such as odds ratio or relative risk per SD difference | 6 | 3.4: Complete MR results with effect estimates |
|  | c) | If relevant, consider translating estimates of relative risk into absolute risk for a meaningful time period |  | Not applicable |
|  | d) | Consider plots to visualize results (e.g. forest plot, scatterplot of associations between genetic variants and outcome versus between genetic variants and exposure) | 6 | Figure 4: Comprehensive MR visualization plots |
| 12 | **Assessment of assumptions** |  | 6 | 3.4: "Sensitivity analyses (Q = 6, P = 0.509) further confirmed the robustness... no evidence of significant heterogeneity or pleiotropic bias" |
|  | a) | Report the assessment of the validity of the assumptions | 6 | 3.4: Heterogeneity and pleiotropy assessments reported |
|  | b) | Report any additional statistics (e.g., assessments of heterogeneity across genetic variants, such as *I^2^*, Q statistic or E-value) | 6 | 3.4: Cochran's Q statistic and P-value provided |
| 13 | **Sensitivity analyses and additional analyses** |  | 6 | 3.4: Consistent direction across MR methods despite some non-significance |
|  | a) | Report any sensitivity analyses to assess the robustness of the main results to violations of the assumptions | 6 | 3.4: Multiple sensitivity analyses support main findings |
|  | b) | Report results from other sensitivity analyses or additional analyses | 6 | 3.4: Leave-one-out and other sensitivity methods |
|  | c) | Report any assessment of direction of causal relationship (e.g., bidirectional MR) |  | Not assessed |
|  | d) | When relevant, report and compare with estimates from non-MR analyses | 6 | *3.5-3.7:* Experimental validation supports MR findings |
|  | e) | Consider additional plots to visualize results (e.g., leave-one-out analyses) | 6-8 | Figure 4: Leave-one-out and other sensitivity plots |
|  | **DISCUSSION** |  |  |  |
| 14 | **Key results** | Summarize key results with reference to study objectives | 9 | *4.1-4.3:* Comprehensive summary of cross-tissue findings and MR results |
| 15 | **Limitations** | Discuss limitations of the study, taking into account the validity of the IV assumptions, other sources of potential bias, and imprecision. Discuss both direction and magnitude of any potential bias and any efforts to address them | 10 | 4.4: Detailed limitations including MR assumptions, population specificity, and functional validation needs |
| 16 | **Interpretation** |  | 9-10 | *4.1-4.4:* Balanced interpretation considering evidence and limitations |
|  | a) | Meaning: Give a cautious overall interpretation of results in the context of their limitations and in comparison with other studies | 9-10 | Discussion: Places findings in context of existing literature |
|  | b) | Mechanism: Discuss underlying biological mechanisms that could drive a potential causal relationship between the investigated exposure and the outcome, and whether the gene-environment equivalence assumption is reasonable. Use causal language carefully, clarifying that IV estimates may provide causal effects only under certain assumptions | 9-10 | *4.2-4.3:* Detailed discussion of TGFBR3 mechanisms |
|  | c) | Clinical relevance: Discuss whether the results have clinical or public policy relevance, and to what extent they inform effect sizes of possible interventions | 9-10 | Discussion: Therapeutic implications mentioned throughout |
| 17 | **Generalizability** | Discuss the generalizability of the study results (a) to other populations, (b) across other exposure periods/timings, and (c) across other levels of exposure | 10 | 4.4: "Mendelian randomization analyses were mainly based on European population eQTL data and need to be validated in ethnic populations" |
|  | **OTHER INFORMATION** |  |  |  |
| 18 | **Funding** | Describe sources of funding and the role of funders in the present study and, if applicable, sources of funding for the databases and original study or studies on which the present study is based | 11 | Funding: Complete funding information provided |
| 19 | **Data and data sharing** | Provide the data used to perform all analyses or report where and how the data can be accessed, and reference these sources in the article. Provide the statistical code needed to reproduce the results in the article, or report whether the code is publicly accessible and if so, where | 5，11 | 2.6 & Availability: Data sharing statements and MR code availability |
| 20 | **Conflicts of Interest** | All authors should declare all potential conflicts of interest | 11 | Competing interests: "The authors declare no competing interests" |

This checklist is copyrighted by the Equator Network under the Creative Commons Attribution 3.0 Unported (CC BY 3.0) license.

1. Skrivankova VW, Richmond RC, Woolf BAR, Yarmolinsky J, Davies NM, Swanson SA, et al. Strengthening the Reporting of Observational Studies in Epidemiology using Mendelian Randomization (STROBE-MR) Statement. JAMA. 2021;under review.

2. Skrivankova VW, Richmond RC, Woolf BAR, Davies NM, Swanson SA, VanderWeele TJ, et al. Strengthening the Reporting of Observational Studies in Epidemiology using Mendelian Randomisation (STROBE-MR): Explanation and Elaboration. BMJ. 2021;375:n2233.
